# Job insecurity and mental health related outcomes among the humanitarian workers during COVID-19 pandemic: a cross-sectional study

**DOI:** 10.1186/s40359-022-00974-7

**Published:** 2022-11-14

**Authors:** Naznin Sultana, Md. Asaduzzaman, Abu Bakkar Siddique, Hafeza Khatun, Farzana Sultana Bari, Md. Nazrul Islam, Arifa Tabassum, Abdus Salam Mondol, Md. Abu Sayem, Abu Yousuf Md Abdullah, M. Pear Hossain, Emmanuel Biracyaza

**Affiliations:** 1 Department of Public Health, North South University, Dhaka, Bangladesh; 2 Department of Public Health Nutrition, Primeasia University, Dhaka, Bangladesh; 3 Maternal and Child Health Division, International Centre for Diarrhoeal Disease Research Bangladesh (icddr,b), Dhaka, Bangladesh; 4 Binary Data Lab, Dhaka, Bangladesh; 5 Department of Community Health and Epidemiology, University of Saskatchewan, Saskatoon, Canada; 6 University Research Co. (URC), Chevy Chase, MD USA; 7 School of Planning, University of Waterloo, Waterloo, ON Canada; 8 Department of Statistics, Bangabandhu Sheikh Mujibur Rahman Science and Technology University, Gopalganj, Bangladesh; 9 Department of Biomedical Sciences, Jockey Club College of Veterinary Medicine and Life Sciences, City University of Hong Kong, Hong Kong, China; 10 Program of Sociotherapy, Prison Fellowship Rwanda (PFR), Kigali, Rwanda

**Keywords:** COVID-19, Job insecurity, Mental health, Depression, Humanitarian worker, Stress

## Abstract

**Background:**

The COVID-19 remains a public health burden that has caused global economic crises, jeopardizing health, jobs, and livelihoods of millions of people around the globe. Several efforts have been made by several countries by implementing several health strategies to attenuate the spread of the pandemic. Although several studies indicated effects of COVID-19 on mental health and its associated factors, very little is known about the underlying mechanism of job insecurity, depression, anxiety, and stress in Bangladesh. Therefore, this study determined the prevalence of job insecurity and depression, anxiety, stress as well as the association between job insecurity, mental health outcomes also contributing determinants amongst humanitarian workers during the COVID-19 pandemic in Bangladesh.

**Methods:**

We conducted a web-based cross-sectional study among 445 humanitarian workers during the COVID-19 pandemic in six sub-districts of Cox’s bazar district of Bangladesh between April and May 2021. The questionnaire was composed of socio-demographic, lifestyle and work related factors. Psychometric instruments like job insecurity scale and depression, anxiety also stress scale (DASS-21) were employed to assess the level of job insecurity and mental health outcomes (depression, anxiety and stress). STATA software version 14 was employed to perform statistical analyses.

**Results:**

The prevalence of job insecurity was 42%. The odds of job insecurity was higher in Kutubdia and Pekua (AOR = 3.1, 95% CI 1.36, 7.22) Teknaf (AOR = 2.9, 95% CI 1.33, 6.41), the impact of dissatisfaction on salary (AOR = 2.3, 95% CI 1.49, 3.58) was evident with job insecurity. The prevalence of moderate to severe depression, anxiety and stress among humanitarian worker were (26%, 7%), (25%, 10%) and (15%, 7%) respectively. Further, the region of work, being female, marital status, work environment, and salary dissatisfaction were contributing factors for poor mental health outcomes. Those with job insecurity were almost 3 times more likely to experience depression (AOR = 2.7, 95% CI 1.85, 4.04), anxiety (AOR = 2.6, 95% CI 1.76, 3.71) and stress (AOR: 2.8; 95% CI 1.89, 4.26), respectively.

**Conclusion:**

Our findings highlight that job security remains essential to help tackle the severity of depression, anxiety and stress in humanitarian workers. The results reflected the critical importance of local and international NGOs addressing poor mental health conditions of their employees to prevent mental health outbreaks.

**Supplementary Information:**

The online version contains supplementary material available at 10.1186/s40359-022-00974-7.

## Background

The novel coronavirus disease 2019 (COVID-19) has caused a global health and economic crisis, jeopardizing the health, jobs, and livelihoods of millions of people around the globe [1]. Many countries have implemented various health strategies to reduce the spread of the COVID-19 virus, including lockdown, closure of non-essential enterprises, and the recommendation to stay and work from home. As a result, numerous productive sectors (from major to small businesses) have been forced to halt operations, resulting in job insecurity, job loss, or temporary layoff of a substantial workforce [2, 3]. Along with the manufacturing, construction, and urban informal economic sectors, the service sector has also been adversely impacted by the COVID-19 pandemic [4]. In particular, the development sector has experienced major funding cuts with decrements in projects, which resulted in severe employment losses [5]. Hence, to understand the nature of job insecurity and its subsequent impact on the mental health-related outcomes among the humanitarian workers in the development sector, this study assessed the prevalence and determinants of job insecurity and its associated impact on mental health-related outcomes amongst the humanitarian workers in Bangladesh during the COVID-19 pandemic.

In this regard, job insecurity is a complex topic or two-dimensional topic to understand, as people around the world may face both qualitative and quantitative forms of job insecurity. Quantitative job insecurity refers to the uncertainty about the survival of the job in the future or relates to the employees about the continued existence of their job in the future whereas qualitative job insecurity is related to the concern of employees about the extent to which their job features may unfavorably change [6]. Preceding studies showed that these dual types of job insecurity can result in negative outcomes or well-being related outcomes that include individual effects (such as mental problems and emotional distresses) and organizational effects (such as lower job satisfaction, lower job commitment, higher turnover intention, reduction of organizational products or productivity) [7]. Further, due to COVID-19, most government and non-government funds had to be diverted from the development projects to help support the general public during lockdowns [8, 9]. For example, the United Kingdom Agency for International Development (UKAID), one of the world’s largest donors, has already declared a £2.9 billion (USD 3.7 billion) reduction of such aid budget. A study showed that around 46% of organizations lost donor funding, with around 60% of non-governmental organizations (NGOs) employees losing their jobs in 2020. In addition, leading NGO (such as Oxfam) have announced job cuts and furloughs in 2020, as the pandemic disrupted income flows and development initiatives [10].

Consequently, workers throughout the world perceived moderate to severe levels of job insecurity. For instance, compared to the non-pandemic phases, a relatively high prevalence of job insecurity was reported in Serbia (30%) and Belgium (21%) [11, 12]. Job insecurity was also observed in USA [13], Australia [14] and Saudi Arabia [15] during COVID-19 pandemic. Similar to other regions, Asia has been equally affected [16]. The development workers in countries like Bangladesh were particularly vulnerable to job insecurity, as much of the development and humanitarian projects in these countries depend on government and foreign funding [17]. Then, the dramatic declines in employment opportunities suggest that significant job uncertainty and financial worries are inevitable [18]. Recent research documented a remarkable increase in mental disorders like anxiety and depression due to job insecurity in the COVID-19 pandemic [19, 20]. These findings are consistent with the results of studies conducted on the economic stressor and mental health of different study populations in Bangladesh [21, 22]. Additionally, past studies reported that job insecurity during the pandemic could be a significant risk factor of mental health disorders for not only the general population but also for humanitarian workers [3, 23–25].

Although job insecurity and mental health disorder during the COVID-19 pandemic have been studied for different sectors in Bangladesh, much of the conclusions about humanitarian workers are derived from the studies conducted in other countries. However, the socioeconomic context of Bangladesh is quite unique, and due to the recent influx of Rohingya refugees in the southeastern part of Bangladesh, a huge humanitarian workforce has been deployed to support the 1.3 million Rohingya refugees [26]. Therefore, to address existing gaps in knowledge on COVID-19 related job insecurity and the subsequent impact on the mental health of humanitarian workers in Bangladesh, this cross-sectional study has set to fulfill three specific objectives:

- Analyze the prevalence and determinants of job insecurity among the humanitarian workers in Bangladesh.
- Assess the prevalence and determinants of job insecurity induced poor mental health outcomes of the humanitarian workers.
- Evaluate the impact of job insecurity on mental health outcomes of the humanitarian workers in Bangladesh.

## Methods

### Study design and participants

A cross-sectional study was conducted among 466 humanitarian workers from different humanitarian organizations of the Cox’s Bazar district in Bangladesh. They were recruited using the convenience sampling. However, as 21 questionnaires presented incomplete data, only 445 respondents were considered in the data analysis. Further, we used convenient sampling to select the respondents because of two specific reasons. First, this study aimed to capture the precise understanding of job insecurity and the perceived mental health conditions of the respondents during the COVID-19 pandemic. Consequently, the study was time-sensitive and required a fast completion to avoid any recall bias. Second, this study did not receive any external funding, and the scope to adopt a more sophisticated sampling procedure was limited by resource constraints. However, non-probability sampling, such as convenience sampling, has been proven to be highly effective in reaching respondents in time-sensitive studies, where the findings do not require generalization beyond the study population [27]. In this regard, convenience sampling perfectly aligned with our primary objective since we aimed to study job insecurity and its associated effect on the mental health of humanitarian workers in Bangladesh.

### Study settings

The present study was conducted in 6 sub-districts (Upazilas) of Cox’s Bazar district in Bangladesh. The sub-districts include Cox’s Bazar Sadar, Ukhiya, Teknaf, Moheshkhali, Kutubdia and Pekua. The Cox’s Bazar is home to 2,289,990 people, covering an area of 2,492 km², and with a sex ratio of 104 [28]. The Cox’s Bazar district was selected because it hosts one of the five world’s largest refugee camps [29]. A total of around 1.2–1.3 million Rohingya refugees or Forcibly Displaced Myanmar Nationals (FDMN) reside in these camps [26, 30]. Thus, about 50 national and international non-governmental organizations are currently operating in this district to provide various services to the local host community and the FDMN [31].

### Data collection procedures

Data collection was carried out from April to May 2021 using a self-reporting questionnaire. Due to COVID-19, an online survey form was created to collect data from the study participants. The online survey link was initially shared among various humanitarian worker groups through popular social media platforms such as Facebook, Messenger, WhatsApp, and LinkedIn. Participants were also requested to share the survey link with their colleagues. In addition, a mailed questionnaire was used to acquire data from participants who were not active on social media.

### Study variables

Based on existing literature, we collected information on four major types of independent and dependent variables: (1) socio-demographic, (2) lifestyle and work (3) job insecurity, and (4) mental health conditions (depression, anxiety, and stress).

### Explanatory variables

#### Socio-demographic

Collected data were age, sex (male and female), monthly income (in BDT/Bangladeshi Taka), marital status, education, and location of the workplace (Cox’s Bazar Sadar, Kutubdia and Pekua, Moheshkhali, Teknaf, Ukhiya). Although Kutubdia and Pekua are two different sub-districts, due to the small number of participants, these two regions were grouped into one category and coded as ‘Kutubdia and Pekua’ during data analysis.

#### Lifestyle and work

Lifestyle variables included smoking, drinking alcohol, and physical exercise. This information was taken as a binary “Yes/No” response. Work-related factors, such as the type of organization, were categorized into international non-governmental organizations (INGO), non-governmental organizations (NGO), and United Nations (UN). The type of contract was classified as permanent or temporary. As the organizations provide services to both the host community and Forcibly Displaced Myanmar Nationals (FDMN), the service types were classified into host community and FDMN.

The job designations were categorized based on job responsibility, such as low, middle, and top levels. Top-level authority was considered as job roles involving the preparation of the organizational objectives, policies, and plans. Contrastingly, the middle-level jobs were considered as those that are directly accountable to top management for the operations of their respective departments and required more commitments to organizational and directional functions. Finally, at the low level, the employee was considered to execute and coordinate day-to-day workflow, which ensures completion of the projects and meeting the deliverable targets [32]. Furthermore, for assessing the working conditions, we asked whether the study participants were satisfied with the working environment and salary. The response was taken as a “Yes/No” response.

### Outcome variables

#### Job insecurity scale (JIS)

JIS is a 4-item psychometric instrument developed by De Witte (2000) and validated by Vander Elst et al. [7]. This instrument is used to assess perceived job insecurity. It is also a global measure of job insecurity that corresponds to our understanding of job insecurity. It includes items referring to the threat or possibility of losing a job and items responding to the fears associated with job loss. The four items in the scale included ‘‘Chances are, I will soon lose my job’’, ‘‘I am sure I can keep my job’’ (reverse coded), ‘‘I feel insecure about the future of my job’’, and ‘‘I think I might lose my job in the near future’’. Respondents were asked to rate these items on a 5-point Likert scale, with 1 representing ‘‘strongly disagree” and 5 referring to ‘‘strongly agree”. The total Scoring was calculated by the items’ sum and then divided by the number of items on the scale, which led to a score between 1 and 5. A higher score from 4–5 was defined as job insecurity, and ‘3’ was considered as the neutral midpoint. In the English version of the JIS, Cronbach’s Alpha was 0.82 [7]. The JIS in our study represented a good internal consistency (Cronbach’s Alpha, α = 0.62) and these findings were supported by the previous studies which documented that the psychometric instrument with an internal consistency of more than 0.6 are categorized to be in acceptable range and then these psychometric instrument can be administered among the respondents [33, 34].

#### Depression, anxiety and stress scale-21 items (DASS-21)

The DASS-21 is a 21-item self-report psychometric instrument used to assess the level of depression, anxiety, and stress. This instrument was modified and simplified based on the original version composed of 42 items or DASS-42 [35]. The scale was previously used to measure the psychiatric comorbidity in different populations, such as psychiatric patients [36], general workers [37], and healthcare workers [38] during COVID-19. This study employed the version of DASS-21 adapted among the population of Bangladesh in order to measure the severity of depression, anxiety, and stress. Then, DASS-21 is a self-reported instrument that has 21 questions with three sub-scales. Each sub-scale has 7 items of depression, anxiety, stress and is based on a four-point Likert scale ranging from 0 (“never”) to 3 (“always”) [35]. The sum scores are derived by adding the scores from each sub-scale of depression, anxiety, and stress and multiplying the sum with two. Therefore, the value of the DASS-21 score lies between 0 and 42. The cut-off scores for depression were: normal (0–9), mild depression (10–13), moderate depression (14–20), severe depression (21–27), and extremely severe depression (28 and above). Similarly, the cut-offs for stress were: normal (0–14), mild stress (15–18), moderate stress (19–25), severe stress (26–33), and extremely severe stress (34 and above).Moreover, the cut-off scores for anxiety were: normal (0–7), mild anxiety (8–9), moderate anxiety (10–14), severe anxiety (15–19), and extremely severe anxiety (20 and above). In the original Bangla version DASS-21, Cronbach’s Alpha values for depression subscales were 0.99 and for anxiety, stress subscales were 0.96 [39]. In the present study, the internal consistency for the depression, stress and anxiety subscales were satisfactory (Alpha of Cronbach, α = 0.79, α=0.77) respectively.

### Statistical analysis

Data were analyzed using the STATA Version.14.1 SE (Stata Corp 2015, College Station, TX, USA). The job insecurity and the mental health outcomes, specifically depression, anxiety, and stress, were treated as separate outcome variables. The association between each of the two outcomes and the socio-demographic characteristics, lifestyle, and work-related variables were assessed using the regression technique. Descriptive statistics such as frequencies and percentages of variables, were calculated to describe socio-demographic characteristics, lifestyle and work-related variables. Further, the unadjusted association between each of the categorical variables with job insecurity and depression, anxiety, also stress were assessed using the Chi-square test. The binary logistic regression model was used to find out the association between job insecurity and relevant exposures. The ordinal logistic regression models were constructed to capture the associations between socio-demographic and other variables with depression, anxiety, also stress levels in the DASS-21 instrument. In both binary and ordinal regression models, known confounders and covariates were adjusted. The exposures were added into the binary or ordinal logistic models if the *p* value ≤ 0.15 in the crude odds ratio estimates. The results were reported using adjusted odds ratios (AOR) and their corresponding 95% confidence intervals (CI). In cases where *p* values were reported, a *p* value less than 0.05 was regarded as significant.

### Ethics

The study was carried out following the ethical standards of the 1964 Helsinki Declaration [40].

The ethical approval for the study was obtained from the ethics committee of the Public Health Nutrition Research Committee of the Department of Public Health Nutrition, Primeasia University (Reference number: PAU/PHN/2021/001). After accepting our online invitation to participate in the study, the respondents were provided with the consent form through a secured and private link. Prior to data collection, the respondents were informed about the study objectives, advantages, risks, and confidentiality of involvement in the study. Then, the written and signed informed consent was obtained from all participants. Confidentiality was respected. The participants were only asked to fill out the research questionnaire if they had consented to participate. All data were stored in a depersonalized and anonymised state.

## Results

The findings suggest that the study participants were primarily located in the Ukhiya region (58.2%), with few respondents from Cox’s Bazar Sadar (6.3%). Most of the respondents were working in non-governmental organizations (58.9%) and were middle-level employees (48.3%). Additionally, more than half of the study participants were female (54.2%), aged between 25 and 30 years (52.8%), and were married (57.3%). When work-related factors were assessed, 51% of the participants were found to be engaged in services related to the FDMN, having completed at least a postgraduate degree (37.1%). Moreover, 90.1% were employed under a temporary contract, and 80.9% were satisfied with their working environment and 63.8% with their salary. About one-third of the study participants belonged to the low-income category (< BDT15000/SD 174). The results of lifestyle-related variables showed that 15.1% were smokers, 5.6% alcohol users, and 82.9% exercised regularly. Further details on the respondents are tabulated in Table 1**.**

**Table 1:** Demographic characteristics of participants in humanitarians (N = 445)

Characteristics	Frequency	Percent
Location		
Cox’s Bazar Sadar	28	6.3
Kutubdia & Pekua	30	6.7
Moheshkhali	95	21.3
Teknaf	33	7.4
Ukhiya	259	58.2
Type of organization		
INGO	171	38.4
NGO	262	58.9
UN	12	2.7
Level of designation		
Low	204	45.8
Middle	215	48.3
Top	26	5.8
Service type		
Forcibly Displaced Myanmar Nationals (FDMN)	227	51
Host Community	218	49
Sex		
Male	204	45.8
Female	241	54.2
Age		
< 25 years	93	20.9
25–30 years	235	52.8
Above 30 years	117	26.3
Marital status		
Married	255	57.3
Others (never married, widowed, divorced)	190	42.7
Education		
Higher secondary	144	32.4
Graduate	117	26.3
Post-graduate	165	37.1
Others	19	4.3
Type of contract (work)		
Permanent	44	9.9
Temporary	401	90.1
Satisfaction on working environment		
Yes	360	80.9
No	85	19.1
Satisfaction on salary		
Yes	284	63.8
No	161	36.2
Monthly income (BDT/USD)		
≤15,000 BDT (USD 174.67)	147	33
15,001–35,000 BDT (USD 174.68–407.57)	101	22.7
35,001–55,000 BDT (USD 407.58-640.46)	98	22
Above 55,000 BDT (above USD 640.46)	99	22.2
Smoking habit		
Yes	67	15.1
No	378	84.9
Alcohol consumption		
Yes	25	5.6
No	420	94.4
Daily physical exercise		
Yes	369	82.9
No	76	17.1

**Fig. 1:**
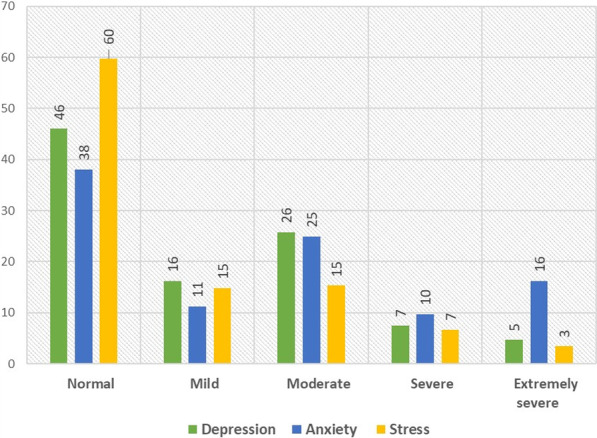
Prevalence of mental health outcomes (depression, anxiety, and stress)

Our result indicated that 42% of study participants suffered from job insecurity. 26%, 7%, and 5% of the humanitarian workers were suffering from moderate, severe, and extremely severe types of depression, respectively. The prevalence of moderate, severe, and extremely severe types of anxiety were 25%, 10%, and 16%, respectively. However, the prevalence of moderate, severe, and extremely severe types of stress was 15%, 7%, and 3%, respectively (Fig. 1).

**Fig. 2:**
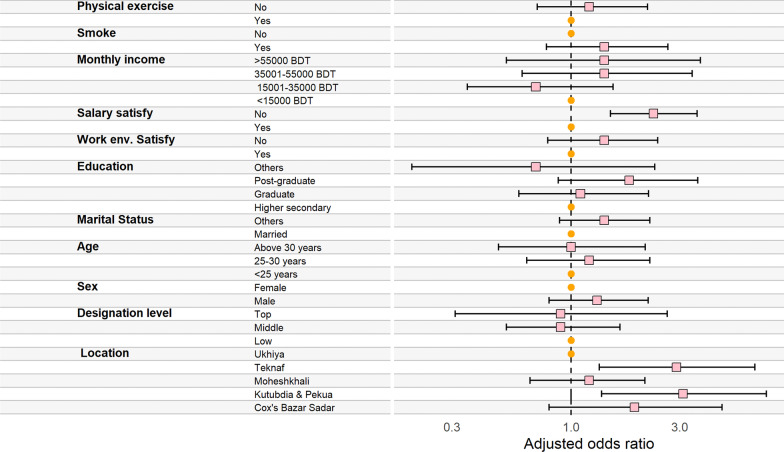
Determinants of job insecurity among the humanitarian worker (Circle: Reference category; square: odds ratio; left and right hand arm of the square: 95% confidence interval)

Figure 2. Reports the determinants of job insecurity among the humanitarian workers. Participants from Kutubdia and Pekua (AOR = 3.1; 95% CI 1.36, 7.22) and Teknaf (AOR = 2.9; 95% CI 1.33, 6.41) were almost three times more likely to experience job insecurity compared to those from Ukhiya. Dissatisfaction with the salary was significantly associated with job insecurity (AOR: 2.3; 95% CI 1.49, 3.58) among the respondents. In individual and unadjusted models, we found that males (UOR: 1.6, 95% CI 1.08, 2.31), completion of post-graduation degree (UOR: 2.0, 95% CI 1.26, 3.16), dissatisfaction with working environment (UOR: 2.0; 95% CI 1.25, 3.25) and smoking (UOR: 2.1; 95% CI 1.25, 3.59) were associated with job insecurity. However, these variables became insignificant when adjusted for potential confounders and covariates (Fig. 2) or see Additional file 1: Table S1.

**Fig. 3:**
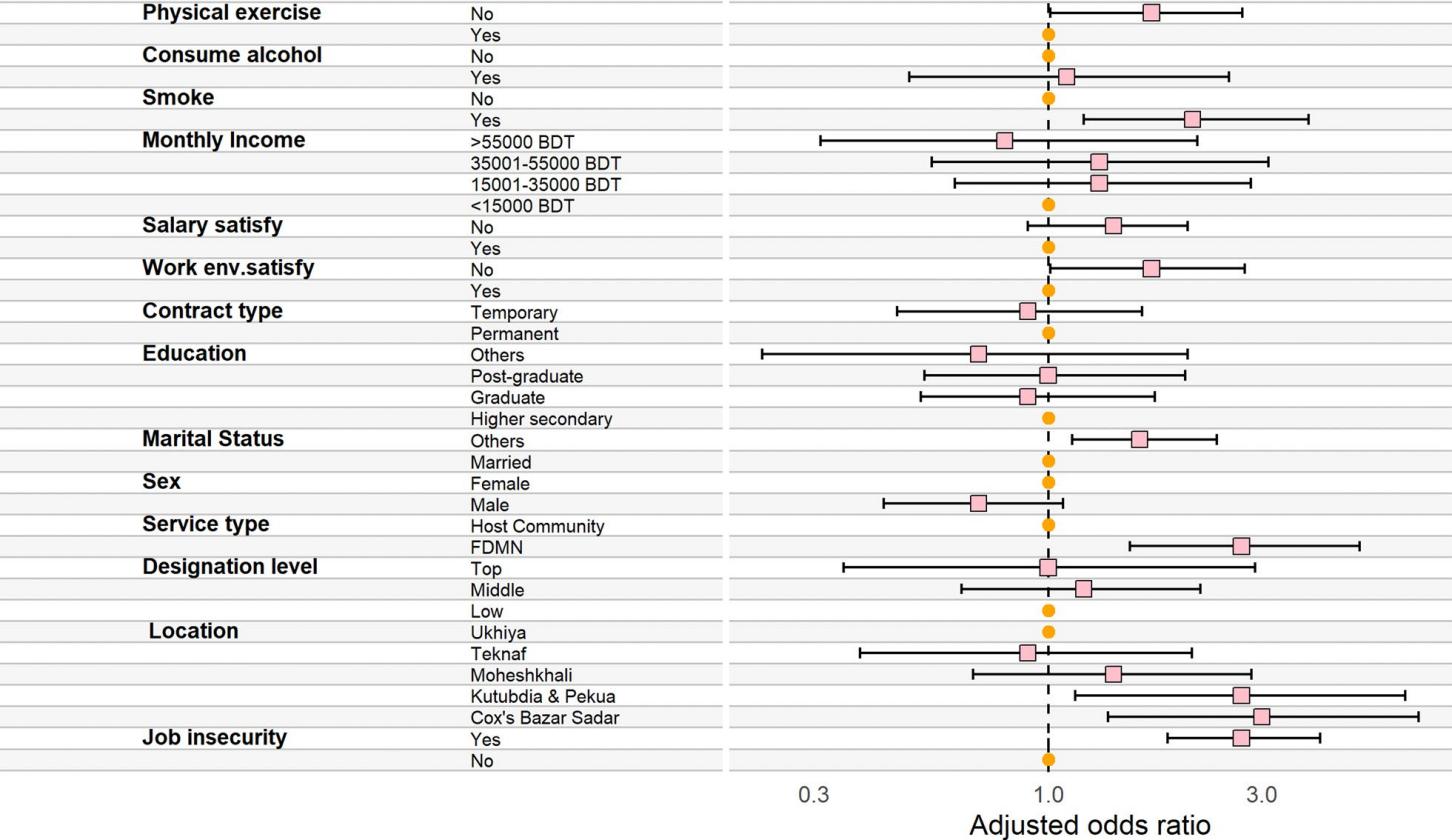
Determinants of depression among the humanitarian worker using ordinal logistic regression (Circle: Reference category; square: odds ratio; left and right hand arm of the square: 95% confidence interval)

Approximately 36.5% and 7.4% of the study participants with job insecurity had suffered from moderate and extremely severe forms of depression (AOR: 2.7; 95% CI 1.85, 4.04). In comparison, only 17.6% and 2.7% of the participants with job insecurity had experienced moderate and extremely severe depression. The respondents who were employed in Cox’s Bazar Sadar (AOR = 3.1; 95% CI 1.36, 6.72), Kutubdia and Pekua (AOR = 2.7; 95% CI 1.15, 6.27) were more likely to be depressed compared to the respondents of Ukhia. Participants who provide services to FDMN were 2.7 (95% CI 1.52, 4.96) times more likely to have mild to extremely severe depression than the respondents who worked with the host community. Regarding marital status, participants who were not married (never married, widowed, divorced) were 1.6 times (95% CI 1.13, 2.38) more likely to be depressed than the married people. Dissatisfaction on workplace environment was associated with higher depression rates (AOR: 1.7; 95% CI 1.01, 2.75) compared with those who were satisfied with their workplace environment. The odds of being depressed were likely to be higher among the participants who smoked daily (AOR: 2.1; 95% CI 1.2, 3.81) and who did not engage in physical exercise (AOR: 1.7; 95% CI 1.01, 2.71) (Fig. 3) or see Additional file 1: Table S2.

**Fig. 4:**
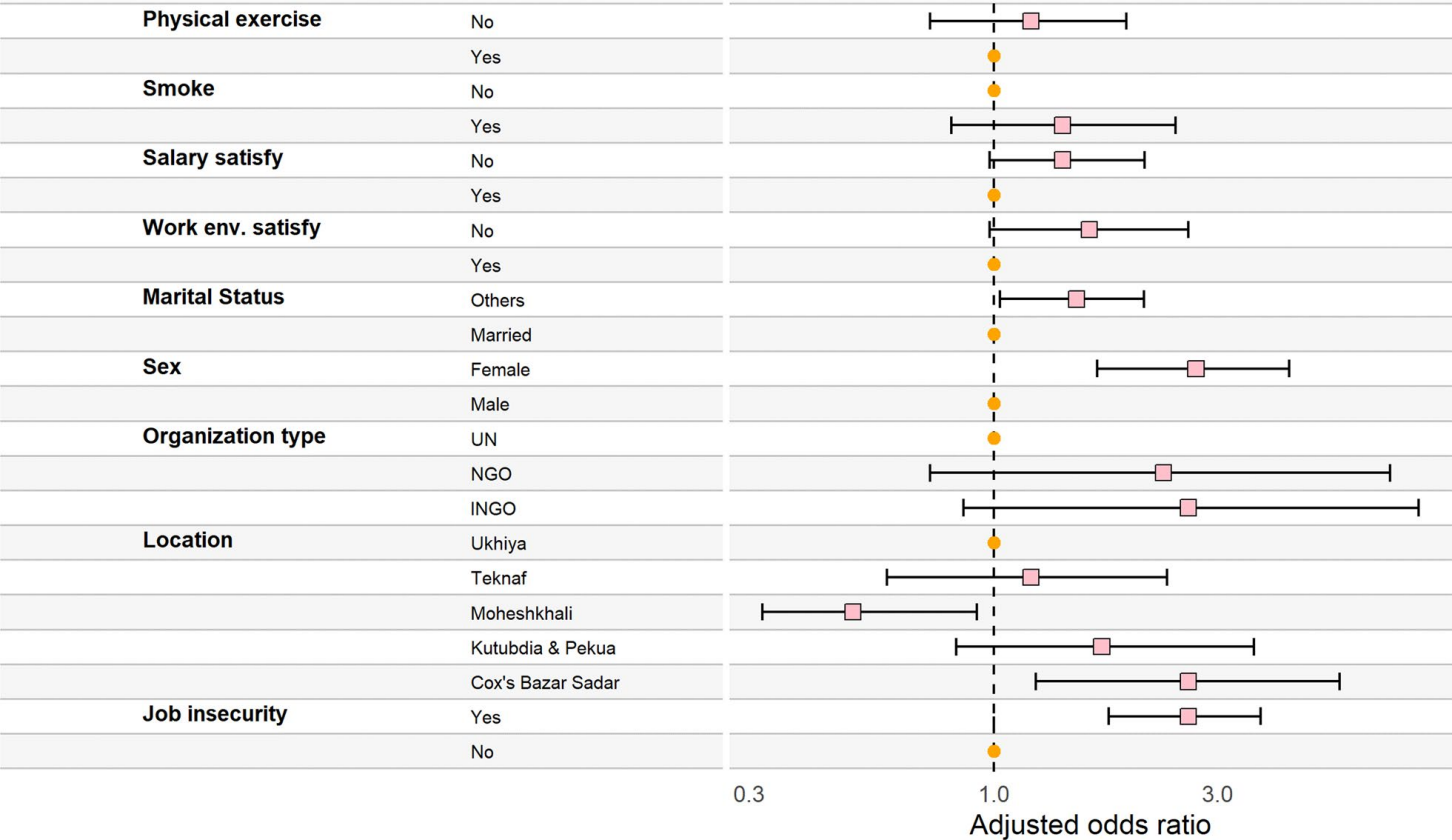
Determinants of anxiety among the Humanitarian Worker using ordinal logistic regression (Circle: Reference category; Square: odds ratio; left and right hand arm of the square: 95% confidence interval)

About half of the humanitarian workers with no job insecurity had experienced a normal anxiety level. In contrast, anxiety was at the normal level for only one-fourth of the participants with job insecurity. Compared to participants without any job insecurity, about 27% of participants with job insecurity reported higher anxiety levels (AOR = 2.6; 95% CI 1.76, 3.71). Respondents who worked in Cox’s Bazar Sadar were almost 3 times more likely to experience anxiety (AOR = 2.6; 95% CI 1.23, 5.48), whereas the respondents who worked in Moheshkhali were least likely (AOR = 0.5; 95% CI 0.32, 0.92) to have any anxiety when compared to their counterparts (Fig. 4) or see Additional file 1: Table S3.

**Fig. 5:**
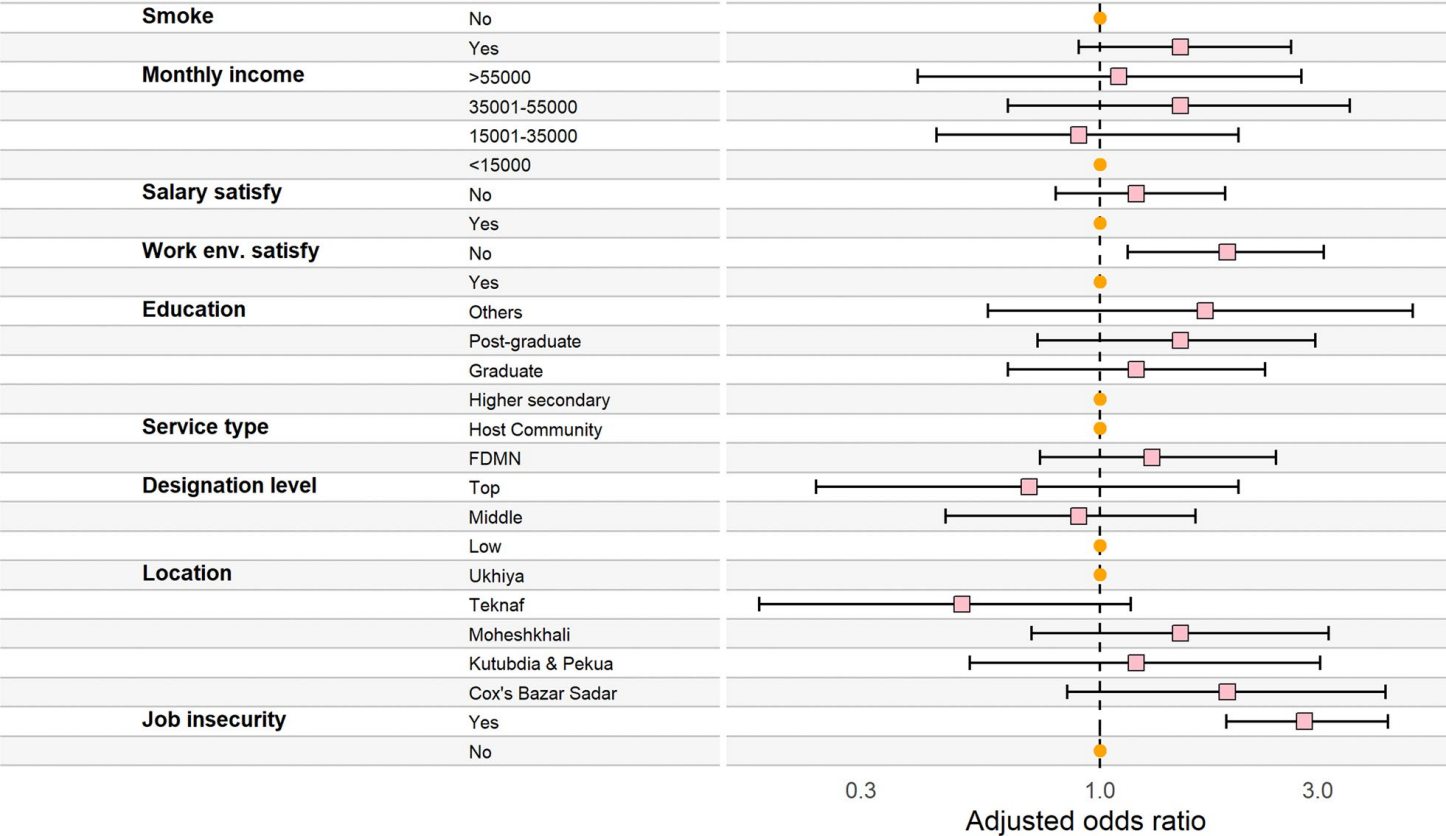
Determinants of stress among the Humanitarian Worker using ordinal logistic regression (Circle: Reference category; Square: Odds ratio; Left and right hand arm of the square: 95% Confidence Interval)

Approximately 20.1%, 11.1% and 6.3% of study participants with job insecurity experienced from moderate, severe and extremely severe stress, respectively (AOR: 2.8; 95% CI 1.89, 4.26), compared to those without job insecurity (11.7%, 3.5 and 1.2%). The location, designation, and educational qualification were significantly associated with stress in the unadjusted models, but no association was observed in the adjusted analyses. Participants who were not satisfied with their working environments were almost twice stressed (AOR = 1.9; 95% CI 1.15, 3.09) compared to the satisfied group (Fig. 5) or see Additional file 1: Table S4.

## Discussion

The major purpose of this study was to determine the prevalence of job insecurity and depression, anxiety, stress. It also assessed the association between job insecurity and mental health outcomes as well as their contributing determinants amongst humanitarian workers during the COVID-19 pandemic in Bangladesh. Further, COVID-19 pandemic causes a tremendous burden on every aspect of human life, including social, economic, and health. It also causes deterioration in mental health conditions among the humanitarian workers in Bangladesh. Due to a substantial reduction in funding, the size of the development and humanitarian projects was reduced during this period, resulting in job losses which increased job insecurity, followed by poor mental health conditions among these workers. This study observed that humanitarian workers with job insecurity were more likely to suffer from adverse mental health outcomes such as depression, anxiety, and stress.

The prevalence of job insecurity among humanitarian workers is substantially high in Bangladesh. The estimated prevalence in this study is 42%, which is consistent with several recent studies. For example, a survey carried out on employed adults from 27 countries reported that 37% are worried to some extent, 54% are worried and 17% are very worried about losing their job [41]. Another study in the United States found the prevalence of mild, moderate, and severe employment insecurity to be 19%, 23%, and 22%, respectively, amongst the already employed individuals [18]. Thus far, there are no prevalence estimates on job insecurity for the humanitarian workers in Bangladesh, especially during the COVID-19 period. Therefore, the findings in this study would guide employers and policymakers to alleviate job insecurity and overcome this mental health-deteriorating burden among the vast humanitarian workforce working in one of the five largest refugee sites in the world [29].

A number of studies suggest COVID-19 is likely to have significant impacts on mental health related outcomes [42–44]. However, the data from the present study revealed that the prevalence of depression (26% & 7%), anxiety (25%, 10%) and stress (15%, 7%) are within the moderate and severe range, respectively. In Bangladesh, there is a lack of comparable statistics on both prevalence and predictors of depression, anxiety, and stress among the humanitarian worker during or before the COVID-19 pandemic. Compared to present study elevated level of depression (33.4%) and stress (44.1%), while lower prevalence of moderate (13.8%) and severe anxiety (2.3%) was observed in 7 eastern African countries (Kenya, Rwandan, Somalia, Sudan, Ethiopia, Tanzanian) among humanitarian and healthcare workers [45]. In the USA, study on employed population observed a lower rate of moderate to severe depression and anxiety (8.9%, 3.6% vs. 16.4%, 8.5%) [18]. Before the COVID-19 pandemic, in South Sudan and Kosover the overall prevalence of depression (39%, 8.6%) and anxiety disorder (38%, 17.1%) was prevalent among humanitarian workers, respectively [46, 47]. The difference in study findings is due to different measurement scales and result of depression, anxiety, stress should be interpreted cautiously.

The present study revealed that participants from Kutubdia & Pekua, Teknaf were suffering more (60%) from job insecurity in comparison to Ukhiya. Maybe the reason is that most of the projects are short-term in these three regions (Kutubdia & Pekua, Teknaf), and a few organizations are working there. A study in Sudan on Humanitarian workers stated that during COVID-19, several organizations have had to shut down entirely due to a lack of funding [48]. As a result, there may be the possibility of chances of job uncertainty among the humanitarian worker. On the other hand, salary acts as a motivation and increase job satisfaction among the worker [49]. In accordance with preceding studies [50, 51], in the current study, job insecurity is significantly associated with salary dissatisfaction.

Participants from Cox’s Bazar Sadar, Kutubdia and Pekua were associated with a higher risk of depressive and anxiety symptoms. A possible cause of these findings is that participants from Kutubdia & Pekua already suffered from high job insecurity. Moreover, most of the participants from Cox’s Bazar Sadar are from managerial positions. Nearly all organizations have offices in Cox’s Bazar Sadar since they need to coordinate with district government offices and their respective sectors. A study from Harvard Business Review stated that supervisors, managers, and middle managers had the highest likelihood of depression and anxiety due to lack of autonomy and work pressure [52]. Participants who worked for the FDMN community were more likely associated with depression than their colleagues who work in the host community. The humanitarian aid sector already operates in a complex environment, and those who work in emergency response are finding it more challenging [53]. FDMN workers suffered more from depression as they witnessed the traumatic event of FDMN community.

Another possible reason for the mental health degradation of humanitarian employees could be due to loneliness caused by not being married or the loss of spouses (divorced or widowed). Studies found that married people are less likely to have mental disorders than those who are single, divorced, or widowed [54]. These findings are consistent with the results we obtained in this study. The present study also showed that not performing physical exercise and smoking were associated with depression. These results are collaborating with the previous studies [45, 55]. In concur with a previous study conducted among humanitarian and healthcare workers reported [45], females from humanitarian settings are more likely to have anxiety than males. Surprisingly, most of our predicting variables do not have any effect on stress except job insecurity and satisfaction with the working environment.

As discussed, job insecurity is associated with adverse mental health outcomes. During the COVID-19 outbreak, many people in low-middle income countries lost jobs and experienced severe financial constraints, increasing the misery in lockstep with employment insecurity [5, 12, 19]. As a result, persistent health, social, and financial insecurity has led to the deterioration of mental strength, followed by mental illness [21, 22, 24, 56, 57]. In concordance with the previous studies, our findings confirm the detrimental impacts of job insecurity on mental health. We observed that people with job insecurity are more likely to suffer from mental illnesses such as depression, anxiety, and stress.

## Strength, limitations and future directions

The strengths of this study stem from its major contributions to the knowledge of challenges faced by humanitarian workers during the COVID-19 pandemic. First, this is the first-ever study in Bangladesh, aiming to assess the prevalence and predictors of job insecurity and mental health-related outcomes (depression, anxiety, and stress). Furthermore, the study explored the potential impacts of job insecurity on depression, anxiety, and stress during the COVID-19 pandemic among the humanitarian workers in Bangladesh. The findings of this study imply that strategies for humanitarian workers should be put into action to mitigate the psychological effects. The results also suggest that the potential long-term implications of job insecurity and the associated mental health complications on these workers should be studied further in detail. Humanitarian employees are the major workforces assisting the devastated and help-seeking population across the world, and therefore, it is imperative that their job insecurities are minimized and mental health promotion initiatives are undertaken.

Despite the significance of the study, there are limitations that need to be considered. First, there is an insufficient number of studies on humanitarian workers in Bangladesh, so we were unable to compare and validate our local-specific results. Second, this study was cross-sectional. Hence, we could not establish causal links between job insecurity and mental health outcomes. Third, data was gathered through self-reported surveys, which results in unavoidable reporting bias. However, the lag between the apex of the COVID-19 outbreak in Bangladesh and the time when this study was conducted is quite small. Therefore, it is reasonable to believe that any reporting and recall bias was not substantial enough to impact the findings adversely. Lastly, we did not look at how respondents’ lifestyles changed during the COVID-19 pandemic or how COVID-19-related factors had affected our dependent measures. For researchers, longitudinal surveys might be incorporated in future studies, which would enable examining whether there is a causal relationship between job insecurity and mental health-related outcomes. Future studies may be conducted on the same issues, and study designs may be improved by incorporating these or other similar variables. It will help us visualize non-pandemic scenarios on job insecurity and mental health-related outcomes of humanitarian workers.

## Conclusion

In summary, our study demonstrated the presence of job insecurity, depression, anxiety, and stress among humanitarian workers during the COVID-19. Factors determined to be associated with job insecurity were salary dissatisfaction and region of the workplace such as Teknaf, Kutubdia, and Pekua. Job insecurity was significantly associated with depression, anxiety and stress. Moreover, depression and anxiety were associated with the working region, sex and marital status. This study highlights the need for mental health assessment and proper management of these issues during the pandemic like COVID-19. These findings call on policymakers to increase job opportunities with the aim of enhancement of job security Also, mental health policies in humanitarian settings need to be prioritized for reducing the magnitude of depression, anxiety and stress .

## Supplementary Information

**Additional file 1**. Table S1. Determinants of job insecurity among the humanitarian worker. Table S2. Determinants of depression among the humanitarian worker using ordinal logistic regression. Table S3. Determinants of anxiety among the Humanitarian Worker using ordinal logistic regression. Table S4. Determinants of stress among the Humanitarian Worker using ordinal logistic regression.

## Data Availability

All relevant data and information can be obtained from the corresponding author upon reasonable request.
