# Supplementary material for: Job insecurity and mental health related outcomes among the humanitarian workers during COVID-19 pandemic: a cross-sectional study
Source: BMC Psychol. 2022 Nov 14;10:265. doi: 10.1186/s40359-022-00974-7 (PMC9660170; doi:10.1186/s40359-022-00974-7)
Supplement: Supplementary file 1 — Additional file 1. Table S1. Determinants of job insecurity among the humanitarian worker. Table S2. Determinants of depression among the humanitarian worker using ordinal logistic regression. Table S3. Determinants of anxiety among the Humanitarian Worker using ordinal logistic regression. Table S4. Determinants of stress among the Humanitarian Worker using ordinal logistic regression. [file 40359_2022_974_MOESM1_ESM.docx]

Table S 1: Determinants of job insecurity among the humanitarian worker

| **Characteristics** | **Job insecurity** | | | **Crude odds ratio** | **Adjusted odds ratio** |
| --- | --- | --- | --- | --- | --- |
|  | **N** | **%** | **p-value** | **COR (95% CI)** | **AOR (95% CI)** |
| Overall | **445** | **42.5** | **-** | **-** | **-** |
| **Location** |  | | | | |
| Cox's Bazar Sadar | 16 | 57.1 |  | 2.0 (0.92,4.44) | 1.9 (0.8,4.61) |
| Kutubdia & Pekua | 18 | 60 |  | **2.3 (1.05,4.91)** | **3.1 (1.36,7.22)** |
| Moheshkhali | 32 | 33.7 | 0.006* | 0.8 (0.47,1.26) | 1.2 (0.66,2.11) |
| Teknaf | 20 | 60.6 |  | **2.3 (1.11,4.89)** | **2.9 (1.33,6.41)** |
| Ukhiya | 103 | 39.8 |  | **ref** | **ref** |
| **Type of organization** |  | | | | |
| INGO | 84 | 49.1 |  | 1.9 (0.56,6.65) |  |
| NGO | 101 | 38.5 | 0.076 | 1.3 (0.37,4.27) |  |
| UN | 4 | 33.3 |  | **ref** |  |
| **Level of Designation** |  | | | | |
| Low | 77 | 37.7 |  | **ref** | **ref** |
| Middle | 100 | 46.5 | 0.179 | 1.4 (0.97,2.12) | 0.9 (0.52,1.64) |
| Top | 12 | 46.2 |  | 1.4 (0.62,3.21) | 0.9 (0.31,2.65) |
| **Service type** |  | | | | |
| Forcibly Displaced Myanmar Nationals (FDMN) | 94 | 41.4 |  | **ref** |  |
| Host Community | 95 | 43.6 | 0.644 | 1.1 (0.75,1.59) |  |
| **Sex** |  | | | | |
| Male | 99 | 48.5 |  | **1.6 (1.08,2.31)** | 1.3 (0.8,2.18) |
| Female | 90 | 37.3 | 0.017* | **ref** | **ref** |
| **Age** |  | | | | |
| <25 years | 34 | 36.6 |  | **ref** | **ref** |
| 25-30 years | 108 | 46 | 0.252 | 1.5 (0.9,2.42) | 1.2 (0.64,2.22) |
| Above 30 years | 47 | 40.2 |  | 1.2 (0.66,2.04) | 1.0 (0.48,2.12) |
| **Marital Status** |  | | | | |
| Married | 100 | 39.2 |  | **ref** | **ref** |
| Others (never married,  widowed, divorced) | 89 | 46.8 | 0.107 | 1.4 (0.93,2) | 1.4 (0.89,2.22) |
| **Education** |  | | | | |
| Higher secondary | 50 | 34.7 |  | **ref** | **ref** |
| Graduate | 49 | 41.9 |  | 1.4 (0.82,2.24) | 1.1 (0.59,2.19) |
| Post-graduate | 85 | 51.5 | 0.011* | **2.0 (1.26,3.16)** | 1.8 (0.88,3.6) |
| Others | 5 | 26.3 |  | 0.7 (0.23,1.97) | 0.7 (0.2,2.33) |
| **Type of contract (work)** |  | | | | |
| Permanent | 20 | 45.5 |  | **ref** |  |
| Temporary | 169 | 42.1 | 0.673 | 0.9 (0.47,1.63) |  |
| **Satisfaction on working environment** |  | | | | |
| Yes | 141 | 39.2 |  | **ref** | **ref** |
| No | 48 | 56.5 | 0.004** | **2.0 (1.25,3.25)** | 1.4 (0.79,2.4) |
| **Satisfaction on Salary** |  |  |  |  |  |
| Yes | 100 | 35.2 |  | **ref** | **ref** |
| No | 89 | 55.3 | <0.001*** | **2.3 (1.53,3.38)** | **2.3 (1.49,3.58)** |
| **Monthly income (BDT)** |  | | | | |
| ≤15000 BDT (USD 174.67) | 53 | 36.1 |  | **ref** | **ref** |
| 15001-35000 BDT (USD 174.68-407.57) | 40 | 39.6 |  | 1.2 (0.69,1.96) | 0.7 (0.35,1.53) |
| 35001-55000 BDT (USD 407.58-640.46) | 48 | 49 | 0.113 | **1.7 (1.01,2.86)** | 1.4 (0.61,3.41) |
| Above 55000 BDT (above USD 640.46) | 48 | 48.5 |  | 1.7 (0.99,2.8) | 1.4 (0.52,3.7) |
| **Smoking habit** |  | | | | |
| Yes | 39 | 58.2 |  | **2.1 (1.25,3.59)** | 1.4 (0.78,2.66) |
| No | 150 | 39.7 | 0.005** | **ref** | **ref** |
| **Alcohol consumption** |  | | | | |
| Yes | 12 | 48 |  | 1.3 (0.56,2.84) |  |
| No | 177 | 42.1 | 0.565 | **ref** |  |
| **Daily physical exercise** |  | | | | |
| Yes | 151 | 40.9 |  | **ref** | **ref** |
| No | 38 | 50 | 0.145 | 1.4 (0.88,2.37) | 1.2 (0.71,2.17) |
| ****: statistical significance at p<0.05*, **: statistical significance at p<0.01 ; ***: statistical significance at p<0.001*** | | | | | |

Table S 2: Determinants of depression among the humanitarian worker using ordinal logistic regression

| **Characteristics** |  | **Normal** | **Mild** | **Moderate** | **Severe** | **Extremely severe** |  | **COR** | **AOR** |
| --- | --- | --- | --- | --- | --- | --- | --- | --- | --- |
|  | N | % | % | % | % | % | **p-value** | **COR (95% CI)** | **AOR (95% CI)** |
| **Job Insecurity** |  |  |  |  |  |  |  |  |  |
| No | 189 | 58.6 | 15.2 | 17.6 | 5.9 | 2.7 |  | ref | ref |
| Yes | 256 | 28.6 | 17.5 | 36.5 | 9.5 | 7.4 | <0.001*** | 3.2 (2.24,4.58) | **2.7 (1.85,4.04)** |
| **Location** |  |  |  |  |  |  |  |  |  |
| Cox's Bazar Sadar | 28 | 17.9 | 28.6 | 32.1 | 14.3 | 7.1 |  | 2 (1.03,3.99) | **3.1 (1.36,6.72)** |
| Kutubdia & Pekua | 30 | 26.7 | 26.7 | 40 | 6.7 | 0 |  | 1.3 (0.68,2.47) | **2.7 (1.15,6.27)** |
| Moheshkhali | 95 | 58.9 | 16.8 | 17.9 | 4.2 | 2.1 | 0.023* | 0.5 (0.32,0.79) | 1.4 (0.68,2.84) |
| Teknaf | 33 | 60.6 | 12.1 | 21.2 | 6.1 | 0 |  | 0.5 (0.24,1) | 0.9 (0.38,2.09) |
| Ukhiya | 259 | 44.4 | 13.9 | 26.6 | 8.1 | 6.6 |  | ref | ref |
| **Type of organization** |  |  |  |  |  |  |  |  |  |
| INGO | 171 | 34.5 | 14 | 35.1 | 9.4 | 6.4 |  | 1.2 (0.47,3.19) |  |
| NGO | 262 | 54.2 | 16.8 | 18.7 | 6.5 | 3.8 | <.01** | 0.5 (0.21,1.4) |  |
| UN | 12 | 25 | 33.3 | 41.7 | 0 | 0 |  | ref |  |
| **Level of Designation** |  |  |  |  |  |  |  |  |  |
| Low | 204 | 52.5 | 16.7 | 20.6 | 6.9 | 3.4 |  | ref | ref |
| Middle | 215 | 40 | 15.3 | 30.7 | 7.9 | 5.6 | 0.365 | 1.7 (1.16,2.36) | 1.2 (0.64,2.19) |
| Top | 26 | 42.3 | 19.2 | 23.1 | 7.7 | 7.7 |  | 1.5 (0.71,3.18) | 1.0 (0.35,2.9) |
| **Service type** |  |  |  |  |  |  |  |  |  |
| Forcibly Displaced Myanmar Nationals (FDMN) | 227 | 38.8 | 14.1 | 30 | 9.3 | 7.5 |  | 2 (1.43,2.87) | **2.7 (1.52,4.96)** |
| Host Community | 218 | 53.2 | 18.3 | 21.1 | 5.5 | 1.8 | <0.01** | ref | ref |
| **Sex** |  |  |  |  |  |  |  |  |  |
| Male | 204 | 42.6 | 15.2 | 27.5 | 10.3 | 4.4 |  | 1.3 (0.95,1.9) | 0.7 (0.43,1.08) |
| Female | 241 | 48.5 | 17 | 24.1 | 5 | 5 | 0.245 | ref | ref |
| **Age** |  |  |  |  |  |  |  |  |  |
| <25 years | 93 | 50.5 | 18.3 | 19.4 | 7.5 | 4.3 |  | ref |  |
| 25-30 years | 235 | 40.9 | 19.1 | 29.8 | 5.1 | 4.7 | 0.056 | 1.4 (0.87,2.11) |  |
| Above 30 years | 117 | 52.1 | 8.5 | 22.2 | 12 | 5.1 |  | 1.2 (0.69,1.94) |  |
| **Marital Status** |  |  |  |  |  |  |  |  |  |
| Married | 255 | 53.3 | 12.5 | 23.1 | 5.9 | 4.7 |  | ref | ref |
| Others (never married ,  widowed, divorced) | 190 | 35.8 | 21.1 | 28.9 | 9.5 | 4.7 | 0.006** | 1.7 (1.22,2.44) | **1.6 (1.13,2.38)** |
| **Education** |  |  |  |  |  |  |  |  |  |
| Higher secondary | 144 | 56.9 | 13.2 | 18.8 | 6.9 | 3.5 |  | ref | ref |
| Graduate | 117 | 40.2 | 22.2 | 25.6 | 6 | 6 |  | 1.7 (1.08,2.69) | 0.9 (0.52,1.73) |
| Post-graduate | 165 | 39.4 | 15.8 | 30.3 | 9.7 | 4.8 | 0.104 | 2 (1.29,3.01) | 1.0 (0.53,2.02) |
| Others | 19 | 52.6 | 5.3 | 36.8 | 0 | 5.3 |  | 1.3 (0.52,3.21) | 0.7 (0.23,2.05) |
| **Type of contract (work)** |  |  |  |  |  |  |  |  |  |
| Permanent | 44 | 36.4 | 11.4 | 34.1 | 11.4 | 4.5 |  | ref | ref |
| Temporary | 401 | 46.9 | 16.7 | 24.7 | 7 | 4.7 | 0.022* | 0.6 (0.36,1.12) | 0.9 (0.46,1.62) |
| **Satisfaction on working environment** |  |  |  |  |  |  |  |  |  |
| Yes | 360 | 49.7 | 17.2 | 23.6 | 6.4 | 3.1 |  | ref | ref |
| No | 85 | 29.4 | 11.8 | 34.1 | 11.8 | 11.8 | <0.001*** | 2.7 (1.76,4.27) | **1.7 (1.01,2.75)** |
| **Satisfaction on Salary** |  |  |  |  |  |  |  |  |  |
| Yes | 284 | 52.5 | 15.1 | 23.2 | 5.6 | 3.5 |  | ref | ref |
| No | 161 | 34.2 | 18 | 29.8 | 10.6 | 6.8 | 0.004** | 2 (1.41,2.89) | 1.4 (0.9,2.05) |
| **Monthly income in BDT** |  |  |  |  |  |  |  |  |  |
| ≤15000 BDT (USD 174.67) | 147 | 56.5 | 17.7 | 17.7 | 6.1 | 2 |  | ref | ref |
| 15001-35000 BDT (USD 174.68-407.57) | 101 | 38.6 | 15.8 | 27.7 | 10.9 | 6.9 |  | 2.2 (1.39,3.61) | 1.3 (0.62,2.83) |
| 35001-55000 BDT (USD 407.58-640.46) | 98 | 38.8 | 13.3 | 31.6 | 7.1 | 8.2 | 0.058 | 2.2 (1.38,3.62) | 1.3 (0.55,3.10) |
| Above 55000 BDT (above USD 640.46) | 99 | 44.4 | 17.2 | 29.3 | 6.1 | 3 |  | 1.6 (0.99,2.56) | 0.8 (0.31,2.15) |
| **Smoking habit** |  |  |  |  |  |  |  |  |  |
| Yes | 67 | 23.9 | 16.4 | 37.3 | 14.9 | 7.5 |  | 2.8 (1.75,4.48) | **2.1 (1.2,3.81)** |
| No | 378 | 49.7 | 16.1 | 23.5 | 6.1 | 4.2 | <.01** | ref | ref |
| **Alcohol consumption** |  |  |  |  |  |  |  |  |  |
| Yes | 25 | 32 | 20 | 20 | 20 | 8 |  | 2 (0.94,4.13) | 1.1 (0.49,2.53) |
| No | 420 | 46.7 | 16 | 26 | 6.7 | 4.5 | 0.157 | ref | ref |
| **Daily physical exercise** |  |  |  |  |  |  |  |  |  |
| Yes | 369 | 49.1 | 16.3 | 24.1 | 7.3 | 3.3 |  | ref | ref |
| No | 76 | 30.3 | 15.8 | 32.9 | 7.9 | 11.8 | <0.01** | 2.2 (1.4,3.49) | **1.7 (1.01,2.71)** |
| ****: statistical significance at p<0.05*, **: statistical significance at p<0.01 ; ***: statistical significance at p<0.001*** | | | | | | | | | |

Table S 3: Determinants of anxiety among the Humanitarian Worker using ordinal logistic regression

| **Characteristics** |  | **Normal** | **Mild** | **Moderate** | **Severe** | **Extremely severe** |  | **COR** | **AOR** | |
| --- | --- | --- | --- | --- | --- | --- | --- | --- | --- | --- |
|  | N | % | % | % | % | % | **p-value** | **COR (95% CI)** | **AOR (95% CI)** | |
| **Job Insecurity** |  |  |  |  |  |  |  |  |  | |
| No | 189 | 48 | 12.5 | 21.5 | 9.8 | 8.2 |  | **ref** | **ref** | |
| Yes | 256 | 24.3 | 9.5 | 29.6 | 9.5 | 27 | <0.001*** | 3 (2.09,4.21) | **2.6 (1.76,3.71)** | |
| **Location** |  |  |  |  |  |  |  |  |  | |
| Cox's Bazar Sadar | 28 | 17.9 | 10.7 | 35.7 | 10.7 | 25 |  | 2 (1.04,4.04) | **2.6 (1.23,5.48)** | |
| Kutubdia & Pekua | 30 | 23.3 | 6.7 | 33.3 | 10 | 26.7 | 0.013* | 2 (1.01,3.86) | 1.7 (0.83,3.59) | |
| Moheshkhali | 95 | 51.6 | 11.6 | 16.8 | 6.3 | 13.7 |  | 0.6 (0.39,0.95) | 0.5 (0.32,0.92) | |
| Teknaf | 33 | 33.3 | 9.1 | 30.3 | 6.1 | 21.2 |  | 1.3 (0.65,2.42) | 1.2 (0.59,2.34) | |
| Ukhiya | 259 | 37.5 | 12 | 25.1 | 11.2 | 14.3 |  | **ref** | **ref** | |
| **Type of organization** |  |  |  |  |  |  |  |  |  | |
| INGO | 171 | 32.7 | 10.5 | 28.7 | 12.3 | 15.8 |  | 2.3 (0.82,6.66) | 2.6 (0.86,8.09) | |
| NGO | 262 | 40.8 | 11.8 | 21.8 | 8.4 | 17.2 | 0.214 | 1.8 (0.64,5.11) | 2.3 (0.73,7.03) | |
| UN | 12 | 50 | 8.3 | 41.7 | 0 | 0 |  | **ref** | **ref** | |
| **Level of Designation** |  |  |  |  |  |  |  |  |  | |
| Low | 204 | 37.3 | 11.3 | 24 | 9.8 | 17.6 |  | **ref** |  | |
| Middle | 215 | 39.5 | 11.2 | 24.7 | 9.8 | 14.9 | 0.975 | 0.9 (0.63,1.26) |  | |
| Top | 26 | 30.8 | 11.5 | 34.6 | 7.7 | 15.4 |  | 1.1 (0.54,2.25) |  | |
| **Service type** |  |  |  |  |  |  |  |  |  | |
| Forcibly Displaced Myanmar Nationals (FDMN) | 227 | 35.7 | 11 | 24.7 | 11.5 | 17.2 |  | 1.2 (0.89,1.74) |  | |
| Host Community | 218 | 40.4 | 11.5 | 25.2 | 7.8 | 15.1 | 0.644 | **ref** |  | |
| **Sex** |  |  |  |  |  |  |  |  |  | |
| Male | 204 | 42.6 | 12.3 | 24 | 7.8 | 13.2 |  | **ref** | **ref** | |
| Female | 241 | 34 | 10.4 | 25.7 | 11.2 | 18.7 | 0.203 | **1.5 (1.09,2.2)** | **2.7 (1.66,4.28)** | |
| **Age** |  |  |  |  |  |  |  |  |  | |
| <25 years | 93 | 34.4 | 12.9 | 23.7 | 11.8 | 17.2 |  | **ref** |  | |
| 25-30 years | 235 | 39.1 | 11.5 | 26.4 | 6.8 | 16.2 | 0.658 | 0.8 (0.54,1.27) |  | |
| Above 30 years | 117 | 38.5 | 9.4 | 23.1 | 13.7 | 15.4 |  | 0.9 (0.56,1.5) |  | |
| **Marital Status** |  |  |  |  |  |  |  |  |  | |
| Married | 255 | 42.4 | 10.6 | 23.5 | 9.8 | 13.7 |  | **ref** | **ref** | |
| Others (never married ,  widowed, divorced) | 190 | 32.1 | 12.1 | 26.8 | 9.5 | 19.5 | 0.203 | 1.5 (1.04,2.04) | **1.5 (1.03,2.09)** | |
| **Education** |  |  |  |  |  |  |  |  |  | |
| Higher secondary | 144 | 41.7 | 13.9 | 19.4 | 9 | 16 |  | **ref** |  | |
| Graduate | 117 | 35 | 9.4 | 27.4 | 11.1 | 17.1 |  | 1.3 (0.85,2.07) |  | |
| Post-graduate | 165 | 37 | 10.3 | 28.5 | 8.5 | 15.8 | 0.885 | 1.2 (0.79,1.77) |  | |
| Others | 19 | 36.8 | 10.5 | 21.1 | 15.8 | 15.8 |  | 1.3 (0.54,3.05) |  | |
| **Type of contract (work)** |  |  |  |  |  |  |  |  |  | |
| Permanent | 44 | 38.6 | 9.1 | 22.7 | 18.2 | 11.4 |  | **ref** |  | |
| Temporary | 401 | 37.9 | 11.5 | 25.2 | 8.7 | 16.7 | 0.323 | 1.0 (0.57,1.73) |  | |
| **Satisfaction on working environment** |  |  |  |  |  |  |  |  |  | |
| Yes | 360 | 40.3 | 11.4 | 26.4 | 7.5 | 14.4 |  |  | **ref** | |
| No | 85 | 28.2 | 10.6 | 18.8 | 18.8 | 23.5 | 0.002** | 1.9 (1.26,2.98) | 1.6 (0.98,2.60) | |
| **Satisfaction on Salary** |  |  |  |  |  |  |  |  |  | |
| Yes | 284 | 43 | 13 | 22.9 | 8.1 | 13 |  | **ref** | **ref** | |
| No | 161 | 29.2 | 8.1 | 28.6 | 12.4 | 21.7 | 0.004* | 1.9 (1.37,2.77) | 1.4 (0.98,2.10) | |
| **Monthly income in BDT** |  |  |  |  |  |  |  |  |  | |
| ≤15000 BDT (USD 174.67) | 147 | 38.1 | 12.2 | 25.2 | 7.5 | 17 |  | **ref** |  | |
| 15001-35000 BDT (USD 174.68-407.57) | 101 | 31.7 | 8.9 | 27.7 | 15.8 | 15.8 |  | 1.3 (0.84,2.08) |  | |
| 35001-55000 BDT (USD 407.58-640.46) | 98 | 40.8 | 12.2 | 19.4 | 8.2 | 19.4 | 0.536 | 1.0 (0.61,1.56) |  | |
| Above 55000 BDT (above USD 640.46) | 99 | 41.4 | 11.1 | 27.3 | 8.1 | 12.1 |  | 0.8 (0.54,1.34) |  | |
| **Smoking habit** |  |  |  |  |  |  |  |  |  | |
| Yes | 67 | 32.8 | 10.4 | 20.9 | 16.4 | 19.4 |  | 1.4 (0.88,2.26) | 1.4 (0.81,2.44) | |
| No | 378 | 38.9 | 11.4 | 25.7 | 8.5 | 15.6 | 0.254 | **ref** | **ref** | |
| **Alcohol consumption** |  |  |  |  |  |  |  |  |  | |
| Yes | 25 | 40 | 12 | 8 | 16 | 24 |  | 1.2 (0.56,2.6) |  | |
| No | 420 | 37.9 | 11.2 | 26 | 9.3 | 15.7 | 0.268 | **ref** |  | |
| **Daily physical exercise** |  |  |  |  |  |  |  |  |  | |
| Yes | 369 | 39.6 | 10.8 | 25.7 | 9.8 | 14.1 |  | **ref** | **ref** | |
| No | 76 | 30.3 | 13.2 | 21.1 | 9.2 | 26.3 | 0.087 | 1.6 (1.03,2.53) | 1.2 (0.73,1.92) | |
| ****: statistical significance at p<0.05*, **: statistical significance at p<0.01 ; ***: statistical significance at p<0.001*** | | | | | | | | | |  |

Table S 4: Determinants of stress among the Humanitarian Worker using ordinal logistic regression

| **Characteristics** |  | **Normal** | **Mild** | **Moderate** | **Severe** | **Extremely severe** |  | **COR** | **AOR** |
| --- | --- | --- | --- | --- | --- | --- | --- | --- | --- |
|  | N | % | % | % | % | % | **p-value** | **COR (95% CI)** | **AOR (95% CI)** |
| **Job Insecurity** |  |  |  |  |  |  |  |  |  |
| No | 189 | 71.1 | 12.5 | 11.7 | 3.5 | 1.2 |  | **ref** | **ref** |
| Yes | 256 | 44.4 | 18 | 20.1 | 11.1 | 6.3 | <0.001*** | 3.2 (2.17,4.62) | **2.8 (1.89,4.26)** |
| **Location** |  |  |  |  |  |  |  |  |  |
| Cox's Bazar Sadar | 28 | 35.7 | 28.6 | 17.9 | 10.7 | 7.1 |  | 2.1 (1.04,4.15) | 1.9 (0.85,4.21) |
| Kutubdia & Pekua | 30 | 50 | 33.3 | 13.3 | 3.3 | 0 |  | 1.1 (0.54,2.1) | 1.2 (0.52,3.03) |
| Moheshkhali | 95 | 67.4 | 6.3 | 9.5 | 13.7 | 3.2 | <0.01** | 0.8 (0.5,1.33) | 1.5 (0.71,3.16) |
| Teknaf | 33 | 75.8 | 12.1 | 9.1 | 3 | 0 |  | 0.4 (0.19,1) | 0.5 (0.18,1.17) |
| Ukhiya | 259 | 58.7 | 14.7 | 18.1 | 4.6 | 3.9 |  | **ref** | **ref** |
| **Type of organization** |  |  |  |  |  |  |  |  |  |
| INGO | 171 | 47.4 | 21.1 | 19.9 | 7 | 4.7 |  | 2.2 (0.68,7.34) |  |
| NGO | 262 | 67.6 | 10.7 | 12.2 | 6.9 | 2.7 | 0.007** | 1.1 (0.34,3.61) |  |
| UN | 12 | 66.7 | 16.7 | 16.7 | 0 | 0 |  | **ref** |  |
| **Level of Designation** |  |  |  |  |  |  |  |  |  |
| Low | 204 | 65.7 | 11.3 | 13.7 | 6.9 | 2.5 |  | **ref** | **ref** |
| Middle | 215 | 55.3 | 16.3 | 16.7 | 7.4 | 4.2 | 0.138 | 1.5 (1.01,2.17) | 0.9 (0.46,1.62) |
| Top | 26 | 50 | 30.8 | 15.4 | 0 | 3.8 |  | 1.4 (0.68,3.02) | 0.7 (0.24,2.01) |
| **Service type** |  |  |  |  |  |  |  |  |  |
| Forcibly Displaced Myanmar Nationals (FDMN) | 227 | 55.1 | 16.7 | 19.4 | 4.4 | 4.4 |  | 1.4 (0.96,2.01) | 1.3 (0.74,2.43) |
| Host Community | 218 | 64.7 | 12.8 | 11 | 9.2 | 2.3 | <0.01** | **ref** | **ref** |
| **Sex** |  |  |  |  |  |  |  |  |  |
| Male | 204 | 58.3 | 18.1 | 17.2 | 4.9 | 1.5 |  | 1.0 (0.67,1.39) |  |
| Female | 241 | 61 | 12 | 13.7 | 8.3 | 5 | 0.046* | **ref** |  |
| **Age** |  |  |  |  |  |  |  |  |  |
| <25 years | 93 | 64.5 | 12.9 | 15.1 | 4.3 | 3.2 |  | **ref** |  |
| 25-30 years | 235 | 58.7 | 15.3 | 16.2 | 6.8 | 3 | 0.943 | 1.3 (0.78,2.04) |  |
| Above 30 years | 117 | 58.1 | 15.4 | 13.7 | 8.5 | 4.3 |  | 1.3 (0.77,2.29) |  |
| **Marital Status** |  |  |  |  |  |  |  |  |  |
| Married | 255 | 62.4 | 15.7 | 11.8 | 6.7 | 3.5 |  | **ref** |  |
| Others (never married,  widowed, divorced) | 190 | 56.3 | 13.7 | 20 | 6.8 | 3.2 | 0.211 | 1.3 (0.9,1.88) |  |
| **Education** |  |  |  |  |  |  |  |  |  |
| Higher secondary | 144 | 68.1 | 13.2 | 13.2 | 4.2 | 1.4 |  | **ref** | **ref** |
| Graduate | 117 | 61.5 | 12 | 13.7 | 7.7 | 5.1 |  | 1.5 (0.88,2.39) | 1.2 (0.63,2.3) |
| Post-graduate | 165 | 51.5 | 18.8 | 17 | 9.1 | 3.6 | 0.186 | 2.0 (1.26,3.07) | 1.5 (0.73,2.96) |
| Others | 19 | 57.9 | 10.5 | 26.3 | 0 | 5.3 |  | 1.6 (0.63,4.09) | 1.7 (0.57,4.84) |
| **Type of contract (work)** |  |  |  |  |  |  |  |  |  |
| Permanent | 44 | 59.1 | 11.4 | 20.5 | 2.3 | 6.8 |  | **ref** |  |
| Temporary | 401 | 59.9 | 15.2 | 14.7 | 7.2 | 3 | 0.352 | 0.9 (0.5,1.7) |  |
| **Satisfaction on working environment** |  |  |  |  |  |  |  |  |  |
| Yes | 360 | 64.2 | 14.2 | 12.8 | 6.4 | 2.5 |  | **ref** | **ref** |
| No | 85 | 41.2 | 17.6 | 25.9 | 8.2 | 7.1 | <0.01** | 2.5 (1.58,3.81) | **1.9 (1.15,3.09)** |
| **Satisfaction on Salary** |  |  |  |  |  |  |  |  |  |
| Yes | 284 | 63 | 16.5 | 13 | 5.3 | 2.1 |  | **ref** | **ref** |
| No | 161 | 54 | 11.8 | 19.3 | 9.3 | 5.6 | 0.020* | 1.6 (1.13,2.4) | 1.2 (0.8,1.88) |
| **Monthly income in BDT** |  |  |  |  |  |  |  |  |  |
| ≤15000 | 147 | 68.7 | 10.2 | 10.9 | 8.2 | 2 |  | **ref** | **ref** |
| 15001-35000 | 101 | 59.4 | 15.8 | 16.8 | 5.9 | 2 |  | 1.4 (0.83,2.3) | 0.9 (0.44,2.01) |
| 35001-55000 | 98 | 53.1 | 16.3 | 14.3 | 8.2 | 8.2 | 0.04* | 1.9 (1.16,3.24) | 1.5 (0.63,3.52) |
| 55000 or more | 99 | 53.5 | 19.2 | 21.2 | 4 | 2 |  | 1.6 (0.99,2.7) | 1.1 (0.4,2.76) |
| **Smoking habit** |  |  |  |  |  |  |  |  |  |
| Yes | 67 | 41.8 | 16.4 | 25.4 | 14.9 | 1.5 |  | 2.3 (1.41,3.66) | 1.5 (0.9,2.62) |
| No | 378 | 63 | 14.6 | 13.5 | 5.3 | 3.7 | <0.01** | **ref** | **ref** |
| **Alcohol consumption** |  |  |  |  |  |  |  |  |  |
| Yes | 25 | 52 | 12 | 20 | 16 | 0 |  | 1.5 (0.69,3.18) |  |
| No | 420 | 60.2 | 15 | 15 | 6.2 | 3.6 | 0.281 | **ref** |  |
| **Daily physical exercise** |  |  |  |  |  |  |  |  |  |
| Yes | 369 | 60.4 | 14.1 | 16.3 | 6.8 | 2.4 |  | **ref** |  |
| No | 76 | 56.6 | 18.4 | 10.5 | 6.6 | 7.9 | 0.096 | 1.2 (0.73,1.92) |  |
| ****: statistical significance at p<0.05*, **: statistical significance at p<0.01 ; ***: statistical significance at p<0.001*** | | | | | | | | | |
